# Cerebrospinal Fluid Biomarkers in Childhood Leukemias

**DOI:** 10.3390/cancers13030438

**Published:** 2021-01-24

**Authors:** Chrysanthy Ikonomidou

**Affiliations:** Department of Neurology, University of Wisconsin Madison, 1685 Highland Avenue, Madison, WI 53705, USA; ikonomidou@neurology.wisc.edu; Tel.: +1-608-2635448

**Keywords:** central nervous system, CNS leukemia, neurotoxicity, synaptic plasticity, cell death, neurocognitive outcome

## Abstract

**Simple Summary:**

In childhood leukemias the central nervous system (CNS) can be invaded by leukemic cells, leading to disease recurrence and treatment failures. Analysis of the cerebrospinal fluid (CSF), which surrounds the brain and spinal cord, is crucial for detection of leukemia in that compartment. Despite advances made in treatments for childhood leukemias, therapies continue to adversely affect the developing brain with resulting neurocognitive deficits in many survivors. This review presents an overview of studies demonstrating that CSF proteins, nucleic acids and metabolites, so called biomarkers, can be utilized in tracing both CNS disease and neurotoxicity of treatments in childhood leukemias.

**Abstract:**

Involvement of the central nervous system (CNS) in childhood leukemias remains a major cause of treatment failures. Analysis of the cerebrospinal fluid constitutes the most important diagnostic pillar in the detection of CNS leukemia and relies primarily on cytological and flow-cytometry studies. With increasing survival rates, it has become clear that treatments for pediatric leukemias pose a toll on the developing brain, as they may cause acute toxicities and persistent neurocognitive deficits. Preclinical research has demonstrated that established and newer therapies can injure and even destroy neuronal and glial cells in the brain. Both passive and active cell death forms can result from DNA damage, oxidative stress, cytokine release, and acceleration of cell aging. In addition, chemotherapy agents may impair neurogenesis as well as the function, formation, and plasticity of synapses. Clinical studies show that neurocognitive toxicity of chemotherapy is greatest in younger children. This raises concerns that, in addition to injury, chemotherapy may also disrupt crucial developmental events resulting in impairment of the formation and efficiency of neuronal networks. This review presents an overview of studies demonstrating that cerebrospinal fluid biomarkers can be utilized in tracing both CNS disease and neurotoxicity of administered treatments in childhood leukemias.

## 1. Introduction

The brain and spinal cord are immersed in cerebrospinal fluid (CSF), which remains in close contact with the interstitial fluid, the leptomeninges, and the choroid plexus, and can be altered in the context of central nervous system (CNS) pathology. CSF hypothesis-driven biomarker research, which has also utilized sophisticated screening multiomics technologies, such as proteomics, metabolomics, and transcriptomics [1], offers unique opportunities for diagnosis, monitoring, and identification of pathways that orchestrate progression of neurologic disease and the impact of therapies on the CNS. CSF biomarker research in childhood leukemia has a long-standing history with a focus on two distinct areas: (1) evaluation for tumor markers indicative of CNS leukemia and (2) biomarkers of brain injury attributable to treatment (chemotherapy, radiation), which may reflect acute neurologic toxicity or processes that may lead to long-term neurocognitive deficits. This review provides a non-exhaustive update on the progress made in this arena.

## 2. CSF Biomarkers Reflecting CNS Disease in Pediatric Patients with Leukemia

Central nervous system involvement in childhood leukemia is rare at initial presentation (3–5%) [2,3,4,5,6,7,8] but much more frequent at relapse with approximately 30–40% of those cases demonstrating CNS disease [3,7]. Advances in cancer treatment have minimized relapses outside the CNS, in particular testicular and extramedullary locations. Nevertheless, CNS relapse still occurs in 3–8% of children with acute lymphoblastic leukemia (ALL) or acute myeloblastic leukemia (AML) [7]. The endothelial blood–brain barrier (BBB), the blood–leptomeningeal barrier (BLMB), and the blood–CSF barrier (BCSFB) are considered most relevant to the migration of leukemic cells into the CNS [8]. A fourth newly identified route is the CNS lymphatic system, which runs within the meninges and along the dural sinuses [9,10,11]. Studies in mice suggest that the BLMB and the BCSFB may be the entry zones for leukemic cells in early stages of the disease [12]. It has also been proposed that leukemia cells may utilize a shortcut along the surface of bridging veins to enter the subarachnoid space [8]. Blast spread within the CNS occurs primarily in the meninges, may persist as focal lesions in the subarachnoid space between arachnoid and pia, and remain undetected by lumbar puncture [8].

Historically, with the improved success in controlling systemic disease, CNS involvement, which underlies the propensity of leukemias to relapse, has moved to the frontline [13]. Predisposing factors of CNS leukemia include high leucocyte counts, T-cell immunophenotype, translocations t(1;19) or t(9;22), and mixed lineage leukemia (*MLL* or *KMT2A*)-rearranged cytogenetics [3,14,15]. In a study by van der Veldenand and colleagues [16], it was shown that CNS leukemic cells have a unique protein expression profile, which differs from that of bone marrow leukemic cells and consists of Stearoyl-CoA desaturase (*SCD*) positivity and increased secreted phosphoprotein 1 or osteopontin (*SPP1*) expression. The presence of this subpopulation of B cell precursor-ALL cells with a “CNS protein profile” in bone marrow aspirates at diagnosis is strongly associated with isolated CNS relapse. Multiple adhesion molecules, chemokines and their receptors, interleukins and their receptors, protein kinases, and growth factors have been implicated in migration, adhesion, and survival of leukemic cells in the CNS [8]. 

The gold standard technology used to evaluate for CNS disease is the detection of leukemic cells in the cerebrospinal fluid (CSF) after lumbar puncture by means of routine cytology [7,8,17]. However, there are multiple factors that hamper the detection of blasts within the CNS, including the small number of cells present in most CSF samples, the presence of contaminating peripheral blood cells, and difficulties in interpreting individual CSF cell morphology in cytospin preparation [18]. 

The crucial observation that most CNS relapses occur in patients initially diagnosed as CNS-negative highlights the urgent need for more sensitive diagnostic and surveillance approaches [18]. Novel strategies aiming to improve sensitivity of CSF diagnosis via qPCR, flow cytometry, analysis of soluble immunological markers, and detection of micro RNAs are being utilized at the preclinical level with promising results (Table 1). These technologies have not yet been adopted in clinical practice, due to methodological challenges and the lack of validation [8,19,20,21]. It should be kept in mind that leukemic blasts may enter the CNS after initiation of treatment. Thus, novel strategies to increase detection sensitivity of cytology will not fully eliminate CNS relapses.

Omans and colleagues [22] used terminal deoxynucleotidyl transferase (TdT) and TdT/CD10 for the staining of CSF cytospin preparations of pediatric patients with ALL. In this study the authors reported that blasts were present in 8% of examined CSF samples, while 33% contained increased numbers of CD10 positive cells but had normal CSF morphology. Thirty-six percent of CSF samples in that study were also examined for the simultaneous presence of nuclear terminal deoxynucleotidy1 transferase (TdT) and demonstrated higher numbers of positive cells for both TdT and CD10. The authors concluded that a large proportion of children with ALL and negative CSF cytology may have CNS disease consistent with the presence of occult leukemic involvement, which can be detected with CD10/TdT staining [22]. 

The yield for detecting leukemic cells within the CSF increases with the use of multi-color flow cytometry utilizing B- and T-cell markers, as demonstrated by several groups of investigators [23,24,25,26]. The detection of subclinical CNS disease by flow cytometry during maintenance correlates with significantly lower 3-year relapse-free survival and 3-year overall survival [24]. It has been suggested that a sensitive methodology like multicolor flow cytometry can be applied for a close follow-up of the levels of leukemic cells in CSF samples, and may identify a group of patients at high risk for relapse.

Using polymerase chain reaction (PCR) with homo/heteroduplex analysis utilizing consensus primers for *IgH* and *TCR* genes, minimal residual disease (MRD) was detected in the CSF in 46.8% of children with ALL with no puncture accident of morphological CNS involvement [27]. All patients included in this study had confirmed *IgH/TCR* gene derangements in bone marrow aspirates. In patients with CNS MRD treated with a less intensive protocol (GBTLI-ALL-93), significantly lower 5-year event-free survival was demonstrated. Similar findings were reported by Scridelli and colleagues [36]. In this study, cytomorphology and IgH/T-cell receptor clonal gene rearrangements, detected by PCR homo/heteroduplex analysis and direct sequencing, were evaluated in CSF free of red blood cells at diagnosis of 37 children with ALL. Molecular CSF involvement was greater as detected by molecular analysis than observed by morphologic criteria (45.9% vs. 5.4%). The 4-year event-free survival was lower in the group with molecularly detected CSF involvement. Next-generation sequencing might offer the potential of even greater sensitivity to detect CNS disease than PCR [37]. 

MicroRNAs (miRs) are highly conserved small non-coding RNAs that are involved in orchestration of proliferation, differentiation, and survival [38]. Altered expression patterns of miRs may be associated with progression of leukemias and other neoplasms [39,40]. In childhood leukemias, miRs can act as oncogenes (miR-29a, miR-125b, miR-143-p3, mir-155, miR-181, miR-183, miR-196b, miR-223) or tumor suppressors (let-7b, miR-29a, miR-99, mir-100, miR-155, miR-181) [40]. They can serve as diagnostic and prognostic biomarkers, which can be used to monitor response to therapy (miR-125b, miR-146b, miR-181c, miR-4786), classification of subgroups (let-7b, miR-98, miR-100, miR-128b, miR-223), and development of new therapeutic agents (mir-10, miR-125b, miR-203, miR-210, miR-335). Favorable prognostic miRNA markers of pediatric leukemias include miR-7, miR-16, miR-33, miR-100, miR-130b, miR-181, miR-215, miR-216, miR-369-5p, miR-496, miR-518d, miR-599, and miR-708. Unfavorable factors include miR-10a, miR-23a, miR-27a, miR-128b, miR-134, miR-150, miR-191, miR-214, miR-223, miR-342, miR-484, miR-486, miR-487, miR-572, miR-580, miR-627, miR-624, let-7g, and let-7i. For a comprehensive review, see reference [40]. 

miRs in childhood leukemias have mainly been studied in blood or bone marrow aspirates. Studies analyzing miRs in the CSF of children with leukemia are scarce. Egyed and colleagues [28] hypothesized that miRs could identify undiagnosed CNS leukemia cases and unravel treatment response in this important compartment. These authors explored miR expression and extracellular vesicle characteristics in CSF of children with acute leukemias. They identified a role of the miR-181 family in clustering CNS-positive and CNS-negative samples and validated miR-181c-5p expression differences between CNS-positive and CNS-negative cases in CSF and bone marrow. The sensitivity of miR-181a measurement in the CSF in childhood ALL highly exceeded those of conventional cytospin in the initial diagnosis of CNS leukemia. Interestingly, measuring miR-181-family levels in peripheral blood samples led to no benefit in diagnosing CNS disease. There was, however, a correlation between bone marrow miR-181a-5p levels and CNS status in patients with precursor B-cell ALL. The investigators concluded that miR-based technologies might provide novel tools to monitor CNS disease in pediatric ALL [28].

Other leukemia-associated factors released into the extracellular space and present in the CSF include soluble L-selectin (sL-selectin) [29], interleukin-2 receptor-α (sIL2-Rα) [30], the chemokine CXCL13 [41], interleukin 7 receptor (IL7R) [31], the chemokine receptor CCR7 [42], monocyte chemotactic protein (CCL2), vascular endothelial growth factor 1 and 2 (VEGF 1 and 2) [32], and osteopontin [33]. 

sIL2Rα is released along with interleukin-2 from activated T-cells. In a study of 77 pediatric patients with ALL, Lee and coinvestigators analyzed both CSF and serum samples [30]. The group reported that discrimination power of CSF sIL2-R for the presence of leukemic blasts was better than that obtained by established cytology techniques. The sensitivity of CSF sIL2-R for detecting CNS disease was 89.5%, and the specificity was 89.6%, whereas the sensitivity and specificity of cytology were 47.4% and 63.2%, respectively. There was no correlation between serum and CSF sIL2-R levels, indicating that sIL2R did not simply diffuse into the CSF compartment from the blood, but rather originated from the leukemic blasts within that compartment. CSF sIL2-R level might therefore be a useful biomarker for CNS leukemia in ALL, especially when combined with conventional cytology and the CSF leukocyte count. 

Vascular endothelial growth factor A (VEGF-A) is highly expressed in solid tumors. It binds to its soluble membrane receptors sVEGFR1 and sVEGFR2. sVEGFRs block tumor promoting activities of VEGF-A. Tang and colleagues investigated sVEGFR1 and R2 as biomarkers in CNS leukemia in CSF and serum samples of 35 patients with ALL with or without CNS disease [32]. sVEGFR1 and sVEGFR2 levels in the CSF of patients with CNS leukemia were 33% higher than in the group without CNS disease. Cox regression analysis showed that CSF levels of sVEGFR2 had a positive effect on event-free survival. Serum levels of sVEGFR1 in the control group were higher than in the CNS-leukemia and non-CNS-leukemia groups, but no difference was found between CNS-leukemia and non-CNS-leukemia patients. Further data analysis suggested that sVEGFR2 CSF levels may be a good predictor for the outcome of leukemia patients [32].

Another potentially interesting biomarker for CNS leukemia is osteopontin, a protein secreted by many cells, including activated T-cells, natural killer cells, and tumor cells. Osteopontin, also known as early T-cell activation gene-1 (SPP1 or ETA-1), is a matricellular protein expressed primarily in the bone marrow. It is produced by osteoblasts, osteoclasts, and other malignant and non-malignant cells and is found as a full-length molecule of transcriptionally processed variants (for a comprehensive review, see [43]). It binds to arginine–glycine–aspartate (RGD) and interacts with integrins. It also interacts with CD44 through non-RGD-mediated mechanisms. By doing so, osteopontin and its transcriptionally processed variants modulate many cellular functions of normal and tumor cells such as adhesion, differentiation, migration, apoptosis, osteogenesis, angiogenesis, tumor growth, dormancy, and extramedullary invasion, including invasion into the CNS. In hematologic malignancies, including pediatric leukemias, osteopontin is generally overexpressed and can serve as a diagnostic and prognostic marker [44,45]. 

The relationship between CSF osteopontin levels and CNS leukemia was evaluated in 62 pediatric patients with acute leukemia and 16 controls [33]. CSF osteopontin levels were higher in patients with leukemia but the difference was not statistically significant. Within the leukemia group, CSF osteopontin levels were higher in the those with CNS disease, but again the difference was not statistically significant. Interestingly, at the time of CNS relapse (detected during follow up), CSF osteopontin levels were significantly higher than in the group with CNS involvement at initial presentation [33]. 

Evaluation of multiplexed biomarkers for assessment of CSF infiltration in pediatric ALL has been attempted for matrix metalloproteinase 9 (MMP-9), monocyte chemotactic protein (CCL-2), soluble vascular cell adhesion molecule-1 (sVCAM-1), interferon gamma (IFN-γ), and inducible protein 10 [34]. An association was found between CNS leukemia, higher sMMP-9, and lower sCCL2 levels in the CSF. In this study, serum biomarker levels were also measured, but no significant differences were found between the groups with and without CNS leukemia.

In addition to the above hypothesis-driven studies, recent advances in proteomics technologies have stimulated interest in the application of mass spectrometry (MS) in qualitative and quantitative analysis of the CSF proteome, in an attempt to better trace the development of CNS leukemia in children and its response to treatment. Advancements in the resolution, mass accuracy, sensitivity and scan rate of mass spectrometers for protein analysis, including label-free and stable isotope labeling approaches, have enabled these types of studies. The label-free strategy injects individual samples directly into the mass spectrometer and quantifies the relative abundance of peptides [46,47,48]. Utilizing comparative proteomic profiling using label-free liquid chromatography–tandem mass spectrometry, Guo and colleagues [35] studied CSF samples from six patients with ALL and six healthy controls. The authors detected 51 differentially expressed proteins, among them two core clusters, including 10 proteins that might be crucial for tumorigenesis and progression of ALL and might potentially be valuable indicators of CNS leukemia. Gene ontology analysis of quantified proteins showed that 47 differentially expressed genes were related to 57 terms in the category of “biological process.” These include cell adhesion, negative regulation of endopeptidase activity, platelet degranulation, signal transduction, receptor-mediated endocytosis, regulation of complement activation, CNS development, regulation of cell growth, complement activation, classical pathway, and axon guidance [35].

**Figure 1:**
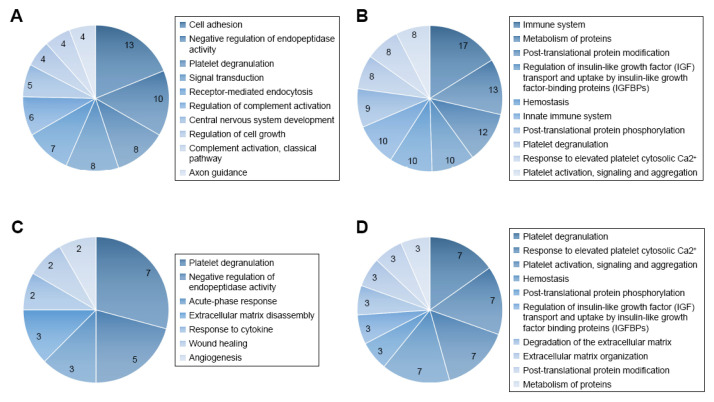
Gene ontology (GO) and REACTOME pathway analysis of quantified proteins. (**A**) Top 10 biological processes obtained from GO analysis of all differentially expressed proteins. (**B**) Top 10 pathways obtained from REACTOME pathway analysis of all differentially expressed proteins. (**C**) Biological processes obtained from GO analysis of cluster 1 proteins. (**D**) Top 10 pathways obtained from REACTOME pathway analysis of cluster 1 proteins. Figure reproduced from Reference [35] with permission from Dove Medical Press, the original publisher.

REACTOME pathway analysis revealed that the differentially expressed genes were associated with a total of 189 pathways (Figure 1). The authors commented that the immune system, cell growth, and platelet function might be essential for pediatric ALL infiltration of the CNS. They also concluded that further analysis of the CSF proteome in ALL could be beneficial in understanding the potential role of selected protein species and biological pathways in the evolution of CNS disease [35].

## 3. Childhood Leukemia Treatment and Brain Toxicity 

Neurocognitive morbidity is frequent in childhood cancer survivors [49,50,51,52,53,54,55,56]. Although neurologic toxicity in childhood leukemias has decreased considerably, mainly due to replacement of cranial irradiation with intrathecal chemotherapy, intellectual development of affected children receiving polychemotherapy can still be compromised [49,50,51,52,53,54,55,56]. 

Lower performance IQ scores compared to controls are most often detected if polychemotherapy is initiated prior to the sixth year of life [57]. Leukoencephalopathies can occur in B-cell ALL survivors many years after the end of successful therapy. Verbal and performance IQs in affected subjects have been reported to lie below 86 [58]. Furthermore, there is a clear correlation between intensity of chemotherapy and neurocognitive deficits [58], which affect visual processing, visual-motor function, attention, concentration, working memory, and executive functions [59,60,61,62,63,64,65].

With the help of preclinical and clinical translational research, knowledge of how chemotherapy impacts the pediatric brain has made significant progress, yet many gaps remain. Modern neuroimaging techniques have helped discover the presence of white (WM) and grey matter (GM) changes, some of which may be reversible and others may persist [66,67,68,69,70,71,72,73]. WM pathologies captured include leukoencephalopathies, volume loss, and reduced fractional anisotropy of white matter tracts within the hippocampus, thalamus, temporal, and frontal lobes [70,71], which are associated with lower IQ [72,73,74,75,76]. The result can be atypical connectome organization, hub connectivity, and reduced cognitive reserve [77,78]. 

Furthermore, adult survivors of ALL were shown to have smaller volumes of grey matter, in particular in the hippocampus, amygdala, thalamus, and nucleus accumbens, which interestingly correlated with lower hippocampal memory performance [68] and smaller surface area in cortical regions, which is associated with problems in executive functioning [79]. Factors that increase the risk for neurotoxicity include young diagnosis age and higher exposure levels to systemic high doses and intrathecal methotrexate [80,81].

## 4. CSF Biomarkers Reflecting Impact of Treatment for Childhood Leukemia on the Central Nervous System

In an attempt to understand how chemotherapy affects the brain and which, if any, CSF biomarkers might help prognosticate neurocognitive outcomes, researchers have used both hypothesis-driven approaches and, more recently, proteomics platforms. Interestingly, changes of selected CSF biomarkers during chemotherapy have been found to correlate with future neurocognitive outcomes (Table 2).

Oesterlundh and colleagues analyzed neurochemical markers of brain injury in CSF during induction treatment for acute ALL in children and reported significant increases in the levels of the neuronal marker neuron-specific-enolase (NSE), the astroglial marker glial fibrillary acidic protein (GFAP), and the axonal marker neurofilament protein light chain (Nfl; marker for axonal injury) [82]. An increase in CSF levels of NSE was already evident during the induction and persisted through the consolidation phase. Others detected elevation of NSE, nerve growth factor (NGF), and brain derived neurotrophic factor (BDNF) in the CSF, proposed to be reflective of neuronal injury [85]. 

In the context of high dose systemic methotrexate treatment in children with ALL, plasma methotrexate levels correlated with increase in the CSF activity of beta-glucuronidase, which could have resulted from leakage of this enzyme from injured or degenerating brain cells [86]. 

Induction chemotherapy may lead to elevated CSF levels of tau, phospho-tau, and neuromodulin. Of these proteins, tau concentrations were found to inversely correlate with future IQ performance [87,88]. CSF tau concentrations were highest in patients who developed leukoencephalopathies [88]. Based on these studies, tau has been proposed as a potential predictive CSF biomarker, which may help identify those ALL survivors with impaired neurocognitive performance [88].

Markers of oxidative stress in the CNS, in particular high concentrations of oxidized CSF phospholipids, were also found to correlate with neurobehavioral problems (aggression, anxiety, somatization, withdrawal, conduct problems, impaired social and leadership skills) [95] and cognitive dysfunction [89], as did folate reductions, homocysteine elevation [90], and increased levels of F2 isoprostanes [96] and caspase 3/7 (apoptosis markers) in patients undergoing intrathecal and high dose systemic methotrexate chemotherapy [91]. In the study by Cole and colleagues [90], serum and erythrocyte folate and homocysteine at initial diagnosis and at the end of each therapy cycle were measured. Higher concentrations of folate were detected in the CSF compared to serum samples. There was a non-significant trend towards lower folate and higher homocysteine levels at diagnosis in both serum and CSF in patients with high risk leukemia relapse compared to those with standard risk. In all patients, CSF folate decreased significantly during induction and throughout the duration of therapy, while CSF homocysteine increased during consolidation phase. A negative correlation was observed between CSF (but not serum) homocysteine at initial diagnosis and the Wechsler Performance IQ during the first month of therapy [90].

In a large study by Cheung and coinvestigators [83], 235 patient CSF samples were assayed at five points, from diagnosis to reinduction, for biomarkers of myelin degradation (myelin basic protein, MBP), neuronal damage (nerve growth factor and total phosphorylated tau protein), astrogliosis (glial fibrillary acidic protein, GFAP), and neuroinflammation (chitotriosidase). MBP and GFAP CSF levels were elevated at baseline and through consolidation. The number of intrathecal injections positively correlated with NGF level increase at consolidation. Increases in GFAP, MBP, and total tau levels were associated with a higher risk for leukoencephalopathy and higher apparent diffusion coefficient in frontal lobe WM 5 years after diagnosis. 

Moore and colleagues [92] analyzed CSF samples from 71 children with ALL, collected at diagnosis and during intrathecal chemotherapy administration. Apoptosis was measured by activity of initiator caspases 8 and 9 and execution caspases 3 and 7. Oxidative stress was assessed by measurements of reduced glutathione (GSH) and oxidized glutathione (GSSG). Low GSH/GSSG ratio was measured and interpreted as indicative of oxidized extracellular environment. The investigators further reported that caspase enzyme activity increased significantly during chemotherapy, and caspases 3 and 7 activity inversely correlated with measures of cognitive abilities, assessed 3 years after ALL diagnosis. 

These hypothesis-driven research studies have demonstrated that cellular injury, oxidative stress, and inflammation occur in the CNS in the context of chemotherapy for childhood leukemias and that CSF biomarkers reflecting these processes may be useful in identifying individuals at risk for worse neurologic outcomes.

In an attempt to discover novel biomarkers that may help trace further pathomechanisms of chemotherapy effects on the brain, proteomics platforms have been utilized. 

**Figure 2:**
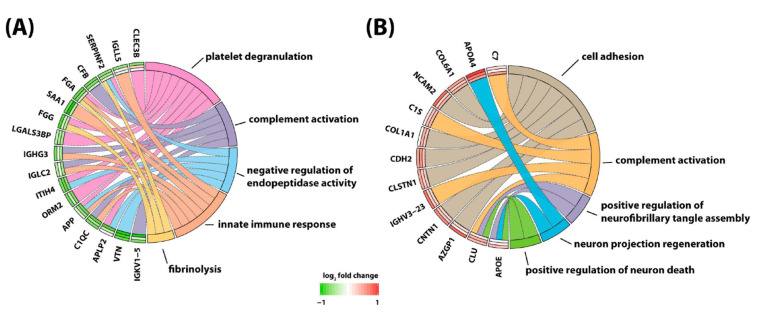
Protein to biological pathway linkages for proteins in two clusters, (**A**) cluster 1 and (**B**) cluster 2, based on hierarchical clustering analysis. The significantly altered proteins (ANOVA, adjusted *p* value < 0.1) are linked to pathways as color-coded ribbons. Green-to-red rectangles next to the altered proteins indicate the magnitude of the log2 fold change (FC), where the inner layer represents the FC of week 1 in comparison with week 5, the middle layer represents the FC of week 1 in comparison with weeks 10−14, and the outer layer represents the FC of week 1 in comparison with weeks 24−28. Figure is reprinted from Yu et al., Isobaric Labeling Strategy Utilizing 4-Plex N, N-Dimethyl Leucine (DiLeu) Tags Reveals Proteomic Changes Induced by Chemotherapy in Cerebrospinal Fluid of Children with B-Cell Acute Lymphoblastic Leukemia. J. Proteome Res. 2020, 19, 2606–2616 [79]. Copyright (2020) American Chemical Society.

Yu and colleagues [93] attempted to globally quantify proteins in CSF of children with B-cell ALL undergoing systemic and CNS directed chemotherapy without irradiation. In a longitudinal study, the researchers implemented a 4-plex N,Ndimethyl leucine (DiLeu) isobaric labeling strategy to investigate protein dynamics. They analyzed CSF samples obtained at weeks 5, 10–14, and 24–28 of chemotherapy. Several differentially expressed proteins were identified. Among them, changes in CSF levels were detected for neural cell adhesion molecule, neuronal growth regulator 1, and secretogranin-3. Interestingly, these three proteins have been associated with the pathophysiology of neurodegenerative diseases. In addition, there was a total of 63 proteins found to be altered at all investigated time points. With the goal to identify potentially modulated biological processes, these proteins were subjected to gene ontology analysis. This analysis revealed involvement of altered proteins in regulation of neuronal death, neuroinflammation, suppression of neurogenesis, microglial activation, neurofibrillary tangle assembly, regulation of endopeptidase activity, and suppression of neurogenesis (Figure 2). Marked elevation of CSF levels of apolipoprotein E (APOE) and clusterin (CLU) were found in the CSF of children receiving chemotherapy for B-cell ALL. Remarkably, these two proteins have also been implicated in the pathogenesis of neurodegenerative dementias [97,98,99,100,101].

**Figure 3:**
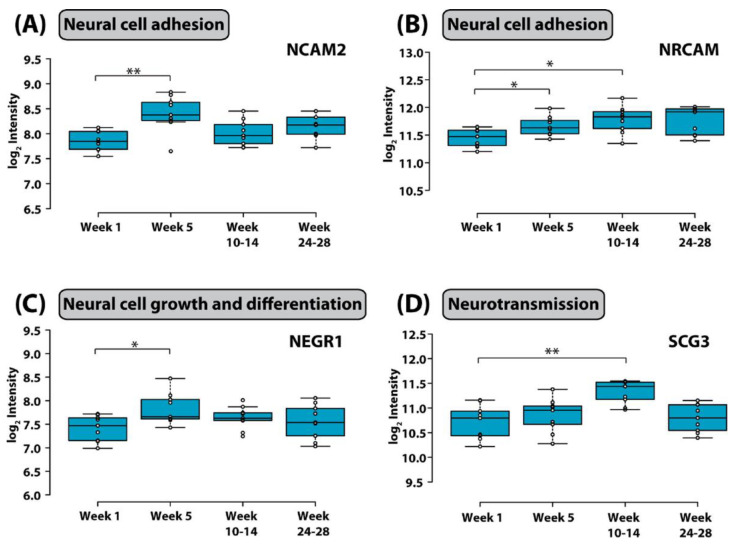
Box plot of the distribution of (**A**) neural cell adhesion molecule 2, (**B**) neuronal cell adhesion molecule, (**C**) neuronal growth regulator 1, and (**D**) secretogranin-3 in CSF in different stages of chemotherapy. Whiskers extend to data points that are less than 1.5* interquartile range away from the first and third quartiles, respectively. The horizontal line shows the median (*, adjusted *p* value < 0.1; ** adjusted *p* value < 0.05, Student’s t test). Figure is reprinted from Yu et al., Isobaric Labeling Strategy Utilizing 4-Plex N, N-Dimethyl Leucine (DiLeu) Tags Reveals Proteomic Changes Induced by Chemotherapy in Cerebrospinal Fluid of Children with B-Cell Acute Lymphoblastic Leukemia. J. Proteome Res. 2020, 19, 2606–2616 [79]. Copyright (2020) American Chemical Society.

Discovering a relationship between identified protein changes in the CSF and pathways that mediate neurotoxic effects of treatment for ALL is a very challenging task. Given that the blood–brain barrier, the blood–CSF barrier and the blood–leptomeningeal barrier can be compromised during cancer chemotherapy and systemic proteins may be allowed to enter the CSF space, alterations measured in the CSF are most likely multifactorial and may reflect consequences of (1) the demise of CNS leukemic cells, (2) the demise of systemic leukemic cells, (3) toxicity of treatment on organs other than the CNS, and (4) toxicity of treatment on the CNS itself. In an attempt to select candidate biomarkers indicative of CNS toxicity, Yu and colleagues identified those proteins that are highly expressed in the brain. Of those, neural cell adhesion molecule 2 (NCAM2) and neuronal cell adhesion molecule (NRCAM), both of which are critical for neural cell adhesion, cell–cell interactions during synaptogenesis, and plasticity [102,103], increased significantly during intensive chemotherapy (Figure 3). NCAM has been found to be elevated in the CSF from patients with mood disorders [104]. Thus, it is possible that chemotherapy in children with ALL may compromise synapse formation and plasticity of neuronal networks.

Neuronal growth regulator 1 (NEGR1) and secretogranin-3 (SCG-3) were also significantly elevated at week 5 and weeks 10–14 respectively. NEGR1 controls neuronal cell growth and differentiation [105], and SCG3 regulates neurotransmitter storage. SCG3 has also been shown to influence apoptosis of dopaminergic neurons [106]. 

Thus, this proteomics approach helped identify interesting protein species that suggest impaired synaptogenesis and synaptic plasticity as new potential mechanisms of chemotherapy-related CNS toxicity. Whether these proteins might represent biomarkers for long-term neurocognitive outcomes will need to be investigated in further studies, which will correlate CSF protein alterations with the results of neuropsychological assessments. 

Brown and colleagues [94] utilized metabolomics technology to explore pathomechanisms of cancer-related fatigue syndrome (CRF). CRF refers to a state of physical and mental exhaustion, which complicates mainly the acute treatment of children with leukemia but can also persist in survivors. The one factor consistently found to be associated with chronic fatigue syndrome in pediatric ALL is exposure to corticosteroids. The pathomechanisms of CRF are poorly understood. During post-induction chemotherapy for ALL, CSF samples were collected from 171 pediatric patients six months after diagnosis. CSF metabolomic profiling was performed using gas chromatography–mass spectrometry (MS) and liquid chromatography–MS/MS. Associations between metabolite abundance and CRF were determined by means of Kendall’s rank correlation. The investigators reported that eight metabolites were significantly associated with fatigue in the discovery cohort. These included gamma-glutamylglutamine peptide, 3-methoxytyrosine, dimethylglycine, asparagine, allantoin, myoinositol, ribitol, and dimethylmalonic acid. These metabolites have been implicated in neurotransmitter transport and glutathione recycling. The authors concluded that impairment of glutamatergic pathways and oxidative stress may contribute to ALL-associated CRF [94].

## 5. Neurotoxicity of Novel Immunotherapies in Childhood Leukemias

CD19-directed chimeric antigen receptor (CAR)-T cell therapy has recently been adopted in the treatment of childhood leukemias [107,108]. After the insertion of a CAR transgene into T-cells in vitro, these cells undergo expansion and are subsequently infused into the patient. The CAR binds to the cancer surface antigen and elicits a cytotoxic cascade, which destroys the malignant cells [109]. Implementation of this novel immunotherapy has revolutionized cancer treatment. However, it has also become increasingly evident that CAR-T-cell treatment can trigger cytokine release syndrome (CRS), a process characterized by profound systemic inflammation and a multitude of neurologic symptoms of variable, potentially life-threatening severity in approximately 40% of patients [110]. The American Society of Bone Marrow Transplantation coined the term immune effector cell-associated neurotoxicity syndrome (ICANS) [111] for this condition, which comprises all neurological toxicities that occur with cell-based immunotherapies. The pathophysiology of ICANS involves disruption of the blood–brain barrier due to the overwhelming systemic inflammation during CAR-T-cell expansion [112,113], whereby monocyte-derived cytokines mediate the development of toxicity [114]. Gust and colleagues [84] reported that CAR-T-cell-mediated neurotoxicity correlated with severity of cytokine release syndrome, abnormal past brain magnetic resonance imaging (MRI), and CAR-T-cell numbers in the blood. Elevation of CSF levels of S100 calcium binding protein B and glial fibrillary acidic protein (GFAP), both astrocytic markers, were detected, suggesting that disruption or destruction of astroglial structure and function underlies the evolution of neurologic symptoms. In this study, corresponding serum GFAP levels did not differ between patients with and without neurotoxicity, and results on serum S100 levels were not presented. CSF white blood cells, total protein levels, interferon-γ (IFNγ), interleukin (IL)-6, IL-10, and granzyme B (GzB) increased in the CSF and correlated with serum levels of IFNγ, IL-10, GzB, granulocyte macrophage colony-stimulating factor, macrophage inflammatory protein 1 alpha, and tumor necrosis factor alpha. Disruption of the neurovascular unit and astrocytic injury were postulated as the mechanism underlying neurologic manifestations of ICANS [84].

## 6. Conclusions

Research on CSF biomarkers for detection of leukemic invasion and for tracing toxicity of treatment in the CNS has experienced remarkable progress in the past 20 years. In addition to a priori hypothesis-driven approaches, technological advances in multiomics methods, in particular sophisticated proteomics platforms, have enabled non-hypothesis-driven analysis of CSF. Insights gained from first pilot studies provide an excellent foundation for further investigations attempting to identify the role of specific proteins and biological pathways in CNS infiltration with leukemic cells on the one hand and, on the other, to understand the pathomechanisms of brain injury caused by the evolving treatments of childhood leukemias.

All biomarkers mentioned in this review are still in the discovery phase. Candidate CSF biomarkers capable of reflecting CNS disease progression, CNS response to therapeutic interventions, or predicting long-term neurocognitive outcomes must undergo a rigorous process of verification, clinical validation for multiple purposes, and multicenter validation for clinical purposes before they can reach the phase of clinical implementation [115]. 

## Figures and Tables

**Table 1:** CSF biomarkers reflecting CNS disease in pediatric patients with leukemia.

Biomarker	Methodology	Significance for CNS Disease	References
Tdt	Immunocytochemistry	Unknown significance for detection of CNS leukemia	[22]
B and T cell markers	Multicolor flow cytometry (FCM)	FCM positive status correlated with CNS disease and shorter overall survival in some studies	[23,24,25,26]
Gene derangements, i.e., *IgH* and *TCR*	PCR with homo/heteroduplex analysis	Clonal IG and/or TCR gene rearrangement in CSF results indicative of minimal residual disease were detected in 46.8% of children with normal CSF cytology	[27]
mi-R-181c-5p	qPCR	mi-R-181a is highly expressed in the CSF of patients with CNS leukemia	[28]
Soluble L-selectin (sl-selectin); *cell adhesion molecule*	ELISA	Elevations are found in CNS leukemia	[29]
Soluble interleukin-2 Receptor-α (sIL2-Rα); *binds interleukin 2*	ELISA	Elevation of sIL2- Rα correlated with CNS disease	[30]
Interleukin 7 receptor (IL7R); *binds ILR7*	Western blot	IL7R is highly expressed in CNS leukemia	[31]
monocyte chemotactic protein (CCL2); *promotes chemotaxis*	ELISA	Unknown significance for detection of CNS leukemia	[32]
Soluble Vascular endothelial growth factor receptor 1&2 (sVEGFR 1 & 2); *Binds VEGFR 1/ 2*	ELISA	Reduction of sVEGFR 2 and sVEGFR2/VEGF ratio was associated with CNS metastasis	[32]
Osteopontin; *matricellular protein expressed in bone marrow*	ELISA	High CSF osteopontin levels correlated with CNS disease	[33]
Matrix metalloproteinase 9 (MMP9); *degrades extracellular matrix*	LUMINEX technology	Higher sMMP-9 levels were found in patients who developed CNS leukemia	[34]
Soluble vascular cell adhesion molecule-1 (sVCAM-1); *adhesion molecule*	LUMINEX technology	Unknown significance for detection of CNS leukemia	[34]
Interferon-γ (IFN-γ);*cytokine*	LUMINEX technology	Unknown significance for detection of CNS leukemia	[34]
Inducible protein 10; *chemokine*	LUMINEX technology	Unknown significance for detection of CNS leukemia	[34]
Differentially expressed proteins (*n* = 51) related to cell adhesion, negative regulation of endopeptidase activity, platelet degranulation, signal transduction, receptor-mediated endocytosis, regulation of comple­ment activation, CNS development, regulation of cell growth, complement activation, classical pathway, and axon guidance	Proteomics technology utilizing label free strategy, mass spectrometry and pathway analysis	Unknown significance for detection of CNS leukemia	[35]

**Table 2:** CSF biomarkers reflecting CNS toxicity of treatment in childhood leukemia.

Biomarker	Methodology	Significance for CNS toxicity of treatment for leukemia	References
Neuron specific enolase (NSE) *Neuronal marker, neuronal injury*	ELISA, radioimmunoassay	Elevations seen during chemotherapy treatment, unknown significance for long term neurocognitive outcome	[82]
GFAP *Astroglial marker, astrogliosis, astroglial injury*	ELISA	Higher CSF levels during chemotherapy associated with higher risk for leukoencephalopathy and higher apparent diffusion coefficient in frontal lobe WM, 5 years after diagnosis. CSF elevations correlate with severity of ICANS-related neurologic symptoms	[82,83,84]
S100 calcium binding protein B *Peptide localized in astrocytes, astrocyte injury*	Magnetic bead assay	Elevated levels correlate with acute neurologic dysfunction CSF elevations correlate with severity of ICANS-related neurologic symptoms	[84]
Neurofilament protein light chain (Nfl) *Neuronal/axonal marker, neuronal/axonal injury*	ELISA	Elevations of CSF levels detected during treatment. Unknown significance for neurocognitive outcome	[82]
Nerve growth factor (NGF); *neurotrophin*	ELISA	Elevations of CSF levels detected during treatment. Unknown significance for neurocognitive outcome	[85]
Brain derived neurotrophic factor (BDNF); *neurotrophin*	ELISA	Elevations of CSF levels detected during treatment. Unknown significance for neurocognitive outcome	[85]
Beta-glucuronidase *Cellular enzyme, cellular injury*	Enzyme activity measurement	Elevations of CSF levels correlate with serum MTX levels Unclear significance for neurocognitive outcome	[86]
Tau; *microtubule-associated* *protein* *expressed in neurons;* *Axonal/neuronal injury*	ELISA	Elevation of CSF levels correlated inversely with IQ performance. Higher CSF levels during chemotherapy associated with higher risk for leukoencephalopathy and higher apparent diffusion coefficient in frontal lobe WM, 5 years after diagnosis	[83,87,88]
Phospho-tau; *phosphorylated tau* *Axonal/neuronal injury*	ELISA	Elevations of CSF levels detected during treatment. Unknown significance for neurocognitive outcome	[83,87,88]
Neuromodulin; *CNS protein involved in axonal growth*	ELISA	Elevations of CSF levels detected during treatment. Unknown significance for neurocognitive outcome	[87]
Oxidized phospholipids; *marker for oxidative stress*	High performance liquid chromatography	Elevations of CSF levels during treatment correlated with cognitive dysfunction assessed 2 years later	[89]
Folate; *cell metabolite; folate physiology*	Radioligand binding assay	Reduction in CSF levels correlated with cognitive dysfunction	[90]
Homocysteine; *accumulates in folate deficiency*	High performance liquid chromatography	Elevations of CSF levels correlated with cognitive dysfunction	[90]
F2 isoprostane; *oxidative stress*	High performance liquid chromatography	Elevations of CSF levels seen in cases of leukoencephalopathy	[91]
Caspase 3/7; *executioner caspases; marker for apoptosis*	Luminescence assay	Elevations of CSF levels correlated with cognitive dysfunction	[91,92]
Myelin basic protein; *marker for oligodendrocytes; myelin injury*	ELISA	Higher CSF levels during chemotherapy associated with higher risk for leukoencephalopathy and higher apparent diffusion coefficient in frontal lobe white matter 5 years after diagnosis	[83]
Chitotriosidase; *enzyme, marks macrophage activation*	Measurement of enzymatic activity	Elevations of CSF levels detected during treatment. Unknown significance for neurocognitive outcome	[83]
Caspase 8 and 9; *initiator caspases*	Luminescence assay	Elevations of CSF levels detected during treatment. Unknown significance for neurocognitive outcome	[92]
reduced: oxidized glutathione GSH:GSSG; *reduction marks oxidative stress*	Luminescence assay	Reductions detected in CSF during treatment. Unknown significance for neurocognitive outcome	[92]
Interferon-γ (IFNγ); *cytokine*	Magnetic bead assay	Elevations are detected in the CSF during ICANS	[84]
interleukin (IL)-6; *cytokine*	Magnetic bead assay	Elevations are detected in the CSF during ICANS	[84]
Interleukin 10 (IL-10); *cytokine*	Magnetic bead assay	Elevations are detected in the CSF during ICANS	[84]
Granzyme B (GzB); *serine protease*	Magnetic bead assay	Elevations are detected in the CSF during ICANS	[84]
Differentially expressed proteins (*n* = 63) involved in regulation of neuronal death, neuroinflammation, suppression of neurogenesis, microglial activation, neurofibrillary tangle assembly, regulation of endopeptidase activity and suppression of neurogenesis	Proteomics technology utilizing a 4-plex N,N dimethyl leucine (DiLeu) isobaric labeling strategy and mass spectroscopy	Unknown significance for neurocognitive outcome	[93]
Differentially expressed metabolites detected in patients with cancer related fatigue syndrome: gamma-glutamylglutamine peptide, 3-methoxytyrosine, dimethylglycine, asparagine, allantoin, myoinositol, ribitol and dimethylmalonic acid	Gas chromatography-Mass spectrometry (MS) and liquid chromatography-MS/MS	Unknown significance for neurocognitive outcome	[94]

## References

[1] Gerner. C., Costigliola V., Golubnitschaja O. (2020). Multiomics patterns in body fluids: Technological challenge with a great potential to implements the advanced paradigm of 3P medicine. Mass. Sec. Rev..

[2] Pui C.-H., Evans W.E. (2006). Treatment of acute lymphoblastic leukemia. N. Eng. J. Med..

[3] Frishman-Levy L., Izraeli S. (2017). Advances in understanding the pathogenesis of CNS acute lymphoblastic leukaemia and potential for therapy. Br. J. Haematol..

[4] Conter V., Arico M., Valsecchi M.G., Basso G., Biondi E., Madon F., Mandelli F., Paolucci G., Pession C., Rizzari C. (2000). Long-term results of the Italian Association of Pediatric Hematology and Oncology (AIEOP) acute lymphoblastic leukemia studies, 1982–1995. Leukemia.

[5] Gaynon P.S., Trigg M.E., Heerema N.A., Sensel M.G., Sather H.N., Hammond G.D., Bleyer W.A. (2000). Children’s Cancer Group trials in childhood acute lymphoblastic leukemia: 1983–1995. Leukemia.

[6] Kamps W.A., Bokkerink J.P., Hakvoort-Cammel F.G., Veerman A.J.P., Weening R.S., van Wering E.R., van Weerden J.F., Hermans J., Slater R., van den Berg E. (2002). BFM-oriented treatment for children with acute lymphoblastic leukemia without cranial irradiation and treatment reduction for standard risk patients: Results of DCLSG protocol ALL-8 (1991–1996). Leukemia.

[7] Pui C.H., Howard S.C. (2008). Current management and challenges of malignant disease in the CNS in pediatric leukemia. Lancet Oncol..

[8] Lenk L., Alsadeq A.l., Schwebe D.M. (2020). Involvement of the central nervous system in acute lymphoblastic leukemia: Opinions on molecular mechanisms and clinical imlications based on recent data. Cancer Met. Rev..

[9] Aspelund A., Antila S., Proulx S.T., Karlsen T.V., Karaman S., Detmar M., Wiig H., Alitalo K. (2015). A dural lymphatic vascular system that drains brain interstitial fluid and macromolecules. J. Exp. Med..

[10] Louveau A., Smirnov I., Keyes T.J., Eccles J.D., Rouhani S.J., Peske J.D., Derecki N.C., Castle D., Mandell J.W., Lee K.S. (2015). Structural and functional features of central nervous system lymphatic vessels. Nature.

[11] Louveau A., Herz J., Alme M.N., Salvador A.F., Dong M.Q., Viar K.E., Herod S.G., Knopp J., Setliff J.C., Lupi A.L. (2018). CNS lymphatic drainage and neuroinflammation are regulated by meningeal lymphatic vasculature. Nat. Neurosci..

[12] Yao H., Price T.T., Cantelli G., Ngo B., Warner M.J., Olivere L., Ridge S.M., Jablonski E.M., Therrien J., Tannheimer S. (2018). Leukaemia hijacks a neural mechanism to invade the central nervous system. Nature.

[13] Silverman L.B., Declerck L., Gelber R.D., Dalton V.K., Asselin B.L., Barr R.D., Clavell L.A., Hurwitz C.A., Moghrabi A., Samson Y. (2000). Results of Dana-Farber Cancer Institute Consortium protocols for children with newly diagnosed acute lymphoblastic leukemia (1981–1995). Leukemia.

[14] Alsadeq A., Schewe D.M. (2017). Acute lymphoblastic leukemia of the central nervous system: On the role of PBX1. Haematologica.

[15] Jeha S., Pei D., Raimondi S.C., Onciu M., Campana D., Cheng C., Sandlund J.T., Ribeiro R.C., Rubnitz J.E., Howard S.C. (2009). Increased risk for CNS relapse in pre-B cell leukemia with the t(1;19)/TCF3-PBX1. Leukemia.

[16] Van der Velden V.H., de Launaij D., de Vries J.F., de Haas V., Sonneveld E., Voerman J.S., de Bie M., Revesz T., Avigad S., Yeoh A.E. (2016). New cellular markers at diagnosis are associated with isolated central nervous system relapse in paediatric B-cell precursor acute lymphoblastic leukaemia. Br. J. Haematol..

[17] Jin M.-W., Xu S.-M., An Q. (2018). Central nervous disease in pediatric patients during acute lymphoblastic leukemia (ALL): A review. Eur. Rev. Med. Pharmacol. Sci..

[18] Bürger B., Zimmermann M., Mann G., Kühl J., Löning L., Riehm H., Reiter A., Schrappe M. (2003). Diagnostic cerebrospinal fluid examination in children with acute lymphoblastic leukemia: Significance of low leukocyte counts with blasts or traumatic lumbar puncture. J. Clin. Oncol..

[19] Del Principe M.I., Maurillo L., Buccisano F., Sconocchia G., Cefalo M., de Santis G., Veroli A.D., Ditto C., Nasso D., Postorino M. (2014). Central nervous system involvement in adult acute lymphoblastic leukemia: Diagnostic tools, prophylaxis, and therapy. Med. J. Hem. Inf. Dis..

[20] Yousafzai Y.M., Smith L., Smith A., Bhatti S., Gardiner M., Cousins A., Fee F., Chudleigh S., Spence A., Taylor W. (2019). Use of quantitative polymerase chain reaction (qPCR) for the diagnosis and monitoring of CNS leukaemia. Leuk. Res..

[21] Thastrup M., Marquart H.V., Levinsen M., Grell K., Abrahamsson J., Albertsen B.K., Frandsen T.L., Harila-Saari A., Lähteenmäki P.M., Niinimäki R. (2020). Flow cytometric detection of leukemic blasts in cerebrospinal fluid predicts risk of relapse in childhood acute lymphoblastic leukemia: A Nordic Society of Pediatric Hematology and Oncology study. Leukemia.

[22] Omans A.C., Barker B.E., Forman E.N., Cornell C.J., Dickerman J.D., Truman J.T. (1990). Immunophenotypic characteristics of cerebrospinal fluid cells in children with acute lymphoblastic leukemia at diagnosis. Blood.

[23] Cancela C.S.P., Murao M., Barcelos J.M., Furtado V.M., Oliveira B.M.D. (2013). Central nervous system involvement in acute lymphoblastic leukemia: Diagnosis by immunophenotyping. J. Bras. Patol. Med. Lab..

[24] Martinez-Laperche C., Gomez-Garcia A.M., Lassaletta A., Moscardo C., Vivanco J.L., Ramirez M. (2013). Detection of occult cerebrospinal fluid involvement during maintenance therapy identifies a group of children with acute lymphoblastic leukemia at high risk for relapse. Am. J. Hematol..

[25] Liang Y., Ca Q., Zhai Z.M., Wang N.L. (2013). A practical strategy of monitoring minimal residue disease and intervention for central nervous system relapse of childhood acute lymphoblastic leukemia: A single Chinese center’s experience. J. Pediatr. Hematol. Oncol..

[26] Ranta S., Nilsson F., Harila-Saari A., Saft L., Tani E., Söderhäll S., Porwit A., Hultdin M., Noren-Nyström U., Heyman M. (2015). Detection of central nervous system involvement in childhood acute lymphoblastic leukemia by cytomorphology and flow cytometry of the cerebrospinal fluid. Pediatr. Blood Cancer.

[27] Biojone E., Queiróz Rde P., Valera E.T., Odashima N.S., Takayanagui O.M., Viana M.B., Tone L.G., Scrideli C.A. (2012). Minimal residual disease in cerebrospinal fluid at diagnosis: A more intensive treatment protocol was able to eliminate the adverse prognosis in children with acute lymphoblastic leukemia. Leuk. Lymphoma.

[28] Egyed B., Kutszegi N., Sági J.C., Gézsi A., Rzepiel A., Visnovitz T., Lőrincz P., Müller J., Zombori M., Szalai C. (2020). MicroRNA-181a as novel liquid biopsy marker of central nervous system involvement in pediatric acute lymphoblastic leukemia. J. Transl. Med..

[29] Stucki A., Cordey A.S., Monai N., Deflaugergues J.C., Schapira H., Spertini O. (1995). Cleaved L-selectin concentrations in meningeal leukemia. Lancet.

[30] Lee W., Kim S.J., Lee S., Kim J., Kim M., Lim J., Kim Y., Cho B., Lee E.J., Han K. (2005). Significance of cerebrospinal fluid sIL-2R level as a marker of CNS involvement in acute lymphoblastic leukemia. Ann. Clin. Lab. Sci..

[31] Alsadeq A., Lenk L., Vadakumchery A., Cousins A., Vokuhl C., Khadour A., Vogiatzu F., Seyfried F., Meyer L.-H., Cario G. (2018). IL7R is associated with CNS infiltration and relapse in pediatric B-cell precursor acute lymphoblastic leukemia. Blood.

[32] Tang Y.T., Jiang F., Guo L., Si M.Y., Jiao X.Y. (2013). The soluble VEGF receptor 1 and 2 expression in cerebral spinal fluid as an indicator for leukemia central nervous system metastasis. J. Neurooncol..

[33] Ncesoy-Özdemir S., ŞAhin G., Bozkurt C., Ören A.C., Balkaya E., Ertem U. (2013). The relationship between cerebrospinal fluid osteopontin level and central nervous system involvement in childhood acute leukemia. Turk. J. Pediatr..

[34] Mikhael N.L., Gendi M., Hassab H., Megahed E.A. (2019). Evaluation of multiplexed biomarkers in assessment of CSF infiltration in pediatric acute lymphoblastic leukemia. Int. J. Hematol. Oncol..

[35] Guo L., Ren H., Zeng H., Gong Y., Ma X. (2019). Proteomic analysis of cerebrospinal fluid in pediatric acute lymphoblastic leukemia patients: A pilot study. OncoTargets Ther..

[36] Scrideli C.A., Queiroz R.P., Takayanagui O.M., Bernardes J.E., Melo E.V., Tone L.G. (2004). Molecular diagnosis of leukemic cerebrospinal fluid cells in children with newly diagnosed acute lymphoblastic leukemia. Haematologica.

[37] Choi J.K., Jeha S., Zheng J., Carlton V., Faham M., Pui C.-H. (2015). Assessment of central nervous system involvement in pediatric acute lymphoblastic leukemia patients using next-generation sequencing method. Blood.

[38] Rupaimoole R., Slack F.J. (2017). MicroRNA therapeutics: Towards a new era for the management of cancer and other diseases. Nat. Rev. Drug Discov..

[39] Lim E.L., Trinh D.L., Ries R.E., Wang J., Gerbing R.B., Ma Y., Topham J., Hughes M., Pleasance E., Mungall A.J. (2017). MicroRNA expression-based model indicates event-free survival in pediatric acute myeloid leukemia. J. Clin. Oncol..

[40] Szcepanke J. (2020). Role of microRNA dysregulation in childhood acute leukemias: Diagnostics, monitoring and therapeutics: A comprehensive review. World J. Clin. Oncol..

[41] Rubenstein J.L., Wong V.S., Kadoch C., Gao H.X., Barajas R., Chen L., Josephson S.A., Lowell C. (2013). Cxcl13 plus interleukin 10 is highly specific for the diagnosis of CNS lymphoma. Blood.

[42] Buonamici S., Trimarchi T., Ruocco M.G., Reavie L., Cathelin S., Mar B.G., Klinakis A., Luyanov Y., Tseng Y., Tseng J.-C. (2009). CCR7 signalling as an essential regulator of CNS infiltration in T-cell leukaemia. Nature.

[43] Bastos A.C.S.F., Blunck C.B., Emerenciano M., Gimba E.R.P. (2017). Osteopontin and their roles in hematological malignancies: Splice variants on the new avenues. Cancer Lett..

[44] Boyerinas B., Zafrir M., Yesilkanal A.E., Price T.T., Hyjek E.M., Sipkins D.A. (2013). Adhesion to osteopontin in the bone marrow niche regulates lymphoblastic leukemia cell dormancy. Blood.

[45] Marroquin C.E., Downey L., Guo H., Kuo P.C. (2004). Osteopontin increases CD44 expression and cell adhesion in RAW 264.7 murine leukemia cells. Immunol. Lett..

[46] Chiang P.K., Lam M.A., Luo Y. (2008). The many faces of amyloid beta in Alzheimer’s disease. Curr. Mol. Med..

[47] Gilchrist A., Au C.E., Hiding J., Bell A.W., Fernandez-Rodriguez J., Lesimple S., Nagaya H., Roy L., Gosline S.J., Hallett M. (2006). Quantitative proteomics analysis of the secretory pathway. Cell.

[48] Cilento E.M., Jin L., Stewart T., Shi M., Sheng L., Zhang J.J. (2019). Mass spectrometry: A platform for biomarker discovery and validation for Alzheimer’s and Parkinson’s diseases. J. Neurochem..

[49] Henderson T.O., Friedman D.L., Meadows A.T. (2010). Childhood cancer survivors: Transition to adult-focused risk-based care. Pediatrics.

[50] Kadan-Lottick N.S., Zeltzer L.K., Liu Q., Yasui Y., Ellenberg L., Gioia G., Robison L.L., Krull K.R. (2010). Neurocognitive functioning in adult survivors of childhood noncentral nervous system cancers. J. Natl. Cancer Inst..

[51] Oeffinger K.C., Nathan P.C., Kremer L.C. (2010). Challenges after curative treatment for childhood cancer and long-term follow up of survivors. Hematol. Oncol. Clin. N. Am..

[52] Zeltzer L.K., Recklitis C., Buchbinder D., Zebrack B., Casillas J., Tsao J.C., Lu Q., Krull K. (2009). Psychological status in childhood cancer survivors: A report from the Childhood Cancer Survivor Study. J. Clin. Oncol..

[53] Hearps S., Seal M., Andersn V., McCarthy M., Connellan M., Downie P., De Luca C. (2017). The relationship between cognitive and neuroimaging outcomes in children treated for acute lymphoblastic leukemia with chemotherapy only: A systematic review. Ped. Blood Cancer.

[54] Krull K.R., Hardy K.K., Kahalley L., Schuitema I., Kesler S.R. (2018). Neurocognitive outcomes and interventons in long-term survivors of childhood cancer. J. Clin. Oncol..

[55] Hardy K.K., Embry L., Kairalla J.A., Helian S., Devidas M., Armstrong D., Hunger S., Carroll W.L., Larsen E., Raetz E.A. (2017). Neurocognitive functioning of children treated for high risk B-acute lymphoblastic leukemia randomly assigned to different methotrexate and corticosteroid treatment strategies: A report from the Children’s Oncology Group. J. Clin. Oncol..

[56] Liu W., Cheung Y.T., Conklin H.M., Jacola L.M., Srivastava D., Nolan V.G., Zhang H., Gurney J.G., Huang I.-C., Robinson L.L. (2018). Evolution of neurocognitive function in long-term survivors of childhood acute lymphoblastic leukemia treated with chemotherapy only. J. Cancer Surv..

[57] Sleurs C., Lemiere J., Vercruysse T., Nolf N., van Calster B., Deprez S., Renard M., Vandecruys E., Benoit Y., Uyttebroeck A. (2017). Intellectual development of childhood ALL patients: A multicenter longitudinal study. Psychooncology.

[58] Duffner P.K., Armstrong F.D., Chen L., Helton K.J., Brecher M.L., Bell B., Chauvenet A.R. (2014). Neurocognitive and neuroradiologic central nervous system late effects in children treated on Pediatric Oncology Group (POG) P9605 (standard risk) and P9201 (lesser risk) acute lymphoblastic leukemia protocols (ACCL0131): A methotrexate consequence? A report from the Children’s Oncology Group. J. Pediatr. Hematol. Oncol..

[59] Anderson F.S., Kunin-Batson A.S. (2009). Neurocognitive late effects of chemotherapy in children: The past 10 years of research on brain structure and function. Pediatr. Blood Cancer.

[60] Ashford J., Schoffstall C., Reddick W.E., Leone C., Laningham F.H., Glass J.O., Pei D., Cheng C., Pui C.H., Conklin H.M. (2010). Attention and working memory abilities in children treated for acute lymphoblastic leukemia. Cancer.

[61] Buizer A.I., de Sonneville L.M., Veerman A.J. (2009). Effects of chemotherapy on neurocognitive function in children with acute lymphoblastic leukemia: A critical review of the literature. Pediatr. Blood Cancer.

[62] Lofstad G.E., Reinfjell T., Hestad K., Diseth T.H. (2009). Cognitive outcome in children and adolescents treated for acute lymphoblastic leukaemia with chemotherapy only. Acta Paediatr..

[63] Moleski M. (2000). Neuropsychological, neuroanatomical, and neurophysiological consequences of CNS chemotherapy for acute lymphoblastic leukemia. Arch. Clin. Neuropsychol..

[64] Von der Weid N., Mosimann I., Hirt A., Wacker P., Nenadov Beck M., Imbach P., Caflisch U., Niggli F., Feldges A., Wagner H.P. (2003). Intellectual outcome in children and adolescents with acute lymphoblastic leukaemia treated with chemotherapy alone: Age- and sex-related differences. Eur. J. Cancer.

[65] Jansen N.C., Kingma A., Schuitema A., Bouma A., Veerman A.J., Kamps W.A. (2008). Neuropsychological outcome in chemotherapy-only-treated children with acute lymphoblastic leukemia. J. Clin. Oncol..

[66] Bhojwani D., Sabin N.D., Pei D., Khan R.B., Panetta J.C., Krull K.R., Inaba H., Rubnitz J.E., Metzger M.L., Howard S.C. (2014). Methotrexate-induced neurotoxicity and leukoencephalopathy in childhood acute lymphoblastic leukemia. J. Clin. Oncol..

[67] Carey M.E., Haut M.W., Reminger S.L., Hutter J.J., Theilmann R., Kaemingk K.L. (2008). Reduced frontal white matter volume in long-term childhood leukemia survivors: A voxel-based morphometry study. Am. J. Neuroradiol..

[68] Genschaft M., Huebner T., Plessow F., Ikonomidou V.N., Abolmaali N., Krone F., Hoffmann A., Holfeld E., Vorwerk P., Kramm C. (2014). Impact of chemotherapy for childhood leukemia on brain morphology and function. PLoS ONE.

[69] Asato R., Akiyama Y., Ito M., Kubota M., Okumura R., Miki Y., Konishi J., Mikawa H. (1992). Nuclear magnetic resonance abnormalities of the cerebral white matter in children with acute lymphoblastic leukemia and malignant lymphoma during and after central nervous system prophylactic treatment with intrathecal methotrexate. Cancer.

[70] Dellani P.R., Eder S., Gawehn J., Fellgiebel A., Müller M.J., Schmidberger H., Stoeter P., Gutjahr P. (2008). Late structural alterations of cerebral white matter in long-term survivors of childhood leukemia. J. Magn. Reson. Imaging.

[71] Van der Plas E., Schachar R.J., Hitzler L., Crosbie J., Guger S.L., Spiegler B.J., Ito S., Nieman B.J. (2016). Brain structure, working memory and response inhibition in childhood leukemia. Brain Behav..

[72] Reddick W.E., Glass J.O., Johnson D.P., Laningham F.H., Pui C.-H. (2009). Voxel-based analysis of T2 hyperintensities in white matter during treatment of childhood leukemia. Am. J. Neuroradiol..

[73] Deprez S., Amant F., Smeets A., Peeters R., Leemans A., Van Hecke W., Verhoeven J.S., Christiaens M.R., Vandenberghe J., Vandenbulcke M. (2012). Longitudinal assessment of chemotherapy-induced changes in cerebral white matter and its correlation with impaired cognitive functioning. J. Clin. Oncol..

[74] Khong P.-L., Leung L.H.T., Fung A.S.M., Fong D.Y.T., Qiu D., Kwong D.L.W., Ooi G.-C., McAlonan G., Cao G., Chan G.C.F. (2006). White matter anisotropy in post-treatment childhood cancer survivors: Preliminary evidence of association with neurocognitive function. J. Clin. Oncol..

[75] Edelmann M.N., Krull K.R., Liu W., Glass J.O., Ji Q., Ogg R.J., Sabin N.D., Srivastava D.K., Robison L.L., Hudson M.M. (2014). Diffusion tenson imaging and neurocognition in survivors of childhood acute lymphoblastic leukaemia. Brain.

[76] ElAlfy M., Ragab I., Azab I., Amin S., Abdel-Maguid M. (2014). Neurocognitive outcome and white matter anisotropy in childhood acute lymphoblastic leukemia survivors treated with different protocols. Pediatr. Hematol. Oncol..

[77] Kesler S.R., Gugel M., Huston-Warren E., Watson C. (2016). Atypical structural connectome organization and cognitive impairment in young survivors of acute lymphoblastic leukemia. Brain Connect..

[78] Sleurs C., Lemiere J., Christiaens D., Billiet T., Peeters R., Sunaert S., Uyttebroeck A., Deprez S. (2018). Advanced MR diffusion imaging and chemotherapy-related changes in cerebral white matter microstructure of survivors of childhood bone and soft tissue sarcoma. Hum. Brain Mapp..

[79] Tamnes C.T., Zeller B., Amlien I.K., Kanellopoulos A., Andersson S., Due- Tønnessen P., Ruud E., Walhovd K.B., Fjell A.M. (2015). Cortical surface area and thickness in adult survivors of pediatric acute lymphoblastic leukemia. Pediatr. Blood Cancer.

[80] Krull K.R., Cheung Y.T., Liu W., Fellah S., Reddick W.E., Brinkman T.M., Kimberg C., Ogg R., Srivastava D., Pui C.H. (2016). Chemotherapy pharmacodynamics and neuroimaging and neurocognitive outcomes in long-term survivors of childhood acute lymphoblastic leukemia. J. Clin. Oncol..

[81] Cheung Y.T., Krull K.R. (2015). Neurocognitive outcomes in long-term survivors of childhood acute lymphoblastic leukemia treated on contemporary treatment protocols: A systematic review. Neurosci. Biobehav. Rev..

[82] Oesterlundh G., Kjellmer I., Lannering B., Rosengren L., Nilsson U.A., Márky I. (2008). Neurochemical markers of brain damage in cerebrospinal fluid during induction treatment of acute lymphoblastic leukemia in children. Pediatr. Blood Cancer.

[83] Cheung Y.T., Khan R.B., Liu W., Brinkman T.M., Edelmann M.N., Reddick W.E., Pei D., Panoskaltsis-Mortari A., Srivastava D., Cheng C. (2018). Association of Cerebrospinal Fluid Biomarkers of Central Nervous System Injury with Neurocognitive and Brain Imaging Outcomes in Children Receiving Chemotherapy for Acute Lymphoblastic Leukemia. JAMA Oncol..

[84] Gust J., Finney O.C., Li D., Hicks R.M., Futrell R.B., Gamble D.N., Rawlings-Rhea S.D., Khalatbari H.K., Ishak G.E., Duncan C.E. (2019). Glial injury in neurotoxicity after pediatric CD19-directed chimeric antigen receptor T cell therapy. Ann. Neurol..

[85] Chiaretti A., Ruggiero A., Coccia P., Antonelli A., Pierri F., Barone G., Attina G., Iuvone L., Maurizi P., Riccardi R. (2011). Expression of liquoral neuroprotection markers in children with acute lymphoblastic leukemia. Leuk. Res..

[86] Viacha V., Eliopoulou M., Haidas S., Beratis N.G. (2004). Correlation of cerebrospinal fluid betal-glucuronidase activity with plasma methotrexate concentrations in leukemic children receiving high-dose methotrexate. Pediatr. Blood Cancer.

[87] Van Gool S.W., De Meyer G., van de Voorde A., Vanmechelen E., Vanderstichele H. (2003). Neurotoxicity marker profiles in the CSF are not age-dependent but show variation in children treated for acute lymphoblastic leukemia. Neurotoxicology.

[88] Krawczuk-Rybak M., Grabowska A., Protal P.T., Muszynska-Roslan K., Braszko J. (2012). Intellectual functioning of childhood leukemia survivors—Relation to Tau protein—A marker of white matter injury. Adv. Med. Sci..

[89] Caron J.E., Krull K.R., Hockenberry M., Jain N., Kaemingk K., Moore I.M. (2009). Oxidative stress and executive function in children receiving chemotherapy for acute lymphoblastic leukemia. Pediatr. Blood Cancer.

[90] Cole P.D., Beckwith K.A., Vijayanathan V., Roychowdhury S., Smith A.K., Kamen B.A. (2009). Folate homeostasis in cerebrospinal fluid during therapy for acute lymphoblastic leukemia. Pediatr. Neurol..

[91] Taylor O.A., Hockenberry M.J., McCarthy K., Gundy P., Montgomery D., Ross A., Scheurer M.E., Moore I.M. (2015). Evaluation of Biomarkeres of Oxidative Stress and Apoptosis in Patients with Severe Methotrexate Neurotoxicity: A case Series. J. Pediatr. Oncol. Nurs..

[92] Moore I.M.K., Koerner K.M., Gundy P.M., Montgomery D.W., Insel K.C., Harris L.L., Taylor O.A., Hockenberry M.J. (2018). Changes in oxidant defense, apoptosis and cognitive abilities during treatment for childhood leukemia. Biol. Res. Nurs..

[93] Yu Q., Zhong X., Chen B., Feng Y., Ma M., Diamond C.A., Voeller J.S., Kim M., DeSantes K.B., Capitini C.M. (2020). Isobaric Labeling Strategy Utilizing 4-Plex *N*, *N*-Dimethyl Leucine (DiLeu) Tags Reveals Proteomic Changes Induced by Chemotherapy in Cerebrospinal Fluid of Children with B-Cell Acute Lymphoblastic Leukemia. J. Proteome Res..

[94] Brown A.L., Sok P., Taylor O., Woodhouse J.P., Bernhardt M.B., Raghubar K.P., Kahalley L.S., Lupo P.J., Hockenberry M.J., Scheuer M.E. (2020). Cerebrospinal Fluid Metabolomic Profiles Associated with Fatigue During Treatment for Pediatric Acute Lymphoblastic Leukemia. J. Pain Symptom Manag..

[95] Stenzel S.L., Krull K.R., Hockenberry M., Jain N., Kaemingk K., Miketova P., Moore I.M. (2010). Oxidative stress and neurobehavioral problems in pediatric acute lymphoblastic leukemia patients undergoing chemotherapy. J. Pediatr. Hematol. Oncol..

[96] Hockenberry M.J., Taylor O.A., Gundy P.M., Ross A.K., Pasvogel A., Montgomery D., Ribbeck P., McCarthy K., Moore I. (2014). F2-Isoprostanes: A measure of oxidative stress in children receiving treatment for leukemia. Biol. Res. Nurs..

[97] Hesse C., Rosengren L., Vanmechelen E., Vanderstichele H., Jensen C., Davidsson P., Blennow K. (2000). Cerebrospinal fluid markers for Alzheimer’s disease evaluated after acute ischemic stroke. J. Alzheimer’s Dis..

[98] Kay A., Petzold A., Kerr M., Keir G., Thompson E., Nicoll J. (2003). Temporal alterations in cerebrospinal fluid amyloid β-protein and apolipoprotein E after subarachnoid hemorrhage. Stroke.

[99] Polihronis M., Paizis K., Carter G., Sedal L., Murphy B. (1993). Elevation of human cerebrospinal fluid clusterin concentration is associated with acute neuropathology. J. Neurol. Sci..

[100] Vranová H.P., Hényková E., Mareš J., Kaiserová M., Menšíková K., Vaštík M., Hluštík P., Zapletalová J., Strnad M., Stejskal D. (2016). Clusterin CSF levels in differential diagnosis of neurodegenerative disorders. J. Neurol. Sci..

[101] Heywood W.E., Galimberti D., Bliss E., Sirka E., Paterson R.W., Magdalinou N.K., Carecchio M., Reid E., Heslegrave A., Fenoglio C. (2015). Identification of novel CSF biomarkers for neurodegeneration and their validation by a high-throughput multiplexed targeted proteomic assay. Mol. Neurodegener..

[102] Rasmussen K.K., Falkesgaard M.H., Winther M., Roed N.K., Quistgaard C.L., Teisen M.N., Edslev S.M., Petersen D.L., Aljubouri A., Christensen C. (2018). NCAM2 Fibronectin type-III domains form a rigid structure that binds and activates the Fibroblast Growth Factor Receptor. Sci. Rep..

[103] Sakurai T. (2012). The role of NrCAM in neural development and disorders—Beyond a simple glue in the brain. Mol. Cell. Neurosci..

[104] Poltorak M., Frye M.A., Wright R., Hemperly J.J., George M.S., Pazzaglia P.J., Jerrels S.A., Post R.M., Freed W.J. (1996). Increased neural cell adhesion molecule in the CSF of patients with mood disorder. J. Neurochem..

[105] Dennis E.L., Jahanshad N., Braskie M.N., Warstadt N.M., Hibar D.P., Kohannim O., Nir T.M., McMahon K.L., de Zubicaray G.I., Montgomery G.W. (2014). Obesity gene NEGR1 associated with white matter integrity in healthy young adults. NeuroImage.

[106] Li F., Tian X., Zhou Y., Zhu L., Wang B., Ding M., Pang H. (2012). Dysregulated expression of secretogranin III is involved in neurotoxin induced dopaminergic neuron apoptosis. J. Neurosci. Res..

[107] Brudno J.N., Kochenderfer J.N. (2018). Chimeric antigen receptor T-cell therapies for lymphoma. Nat. Rev. Clin. Oncol..

[108] Annesley C.E., Summers C., Ceppi F., Gardner R.A. (2018). The evolution and future of CAR T cells for B-cell acute lymphoblastic leukemia. Clin. Pharm. Ther..

[109] Fesnak A.D., June C.H., Levine B.L. (2016). Engineered T cells: The promise and challenges of cancer immunotherapy. Nat. Rev. Cancer.

[110] Gust J., Taraseviciute A., Turtle C.J. (2018). Neurotoxicity associated with CD19-targeted CAR-T cell therapies. CNS Drugs.

[111] Lee D.W., Santomasso B.D., Locke F.L., Ghobadi A., Turtle C.J., Brudno J.N., Maus M.V., Park J.H., Mead E., Pavletic S. (2019). ASBMT consensus grading for cytokine release syndrome and neurological toxicity associated with immune effector cells. Biol. Blood Marrow Transpl..

[112] Taraseviciute A., Tkachev V., Ponce R., Turtle C.J., Snyder J.M., Liggitt H.D., Myerson D., Gonzalez-Cuyar L., Baldessari A., English C. (2018). Chimeric antigen receptor T cell-mediated neurotoxicity in non-human primates. Cancer Discov..

[113] Gust J., Hay K.A., Hanafi L.A., Li D., Myerson D., Gonzalez-Cuyar L.F., Yeung C., Liles C., Wurfel M., Lopez J.A. (2017). Endothelial activation and bloodbrain barrier disruption in neurotoxicity after adoptive immunotherapy with CD19 CAR-T cells. Cancer Discov..

[114] Norelli M., Camisa B., Barbiera G., Falcone L., Purevdori A., Genua M., Sanvito F., Ponzoni M., Doglioni C., Cristofori P. (2018). Monocyte-derived IL-1 and IL-6 are differentially required for cytokine-release syndrome and neurotoxicity due to CAR T cells. Nat. Med..

[115] Teunissen C.E., Verheul C., Willemse E.A.J. (2018). The use of cerebrospinal fluid in biomarker studies. Handb. Clin. Neurol..

